# Teacher-student relationship, coping style and sports anxiety among middle school students: a situational study based in Shanghai

**DOI:** 10.3389/fpsyg.2025.1541580

**Published:** 2025-02-21

**Authors:** Jiatian Qi, Jingjing Li, Bing Liu

**Affiliations:** ^1^Physical Education College, Shanghai University, Shanghai, China; ^2^School of Economics, Shanghai University, Shanghai, China; ^3^Sports Science Research Center, Shanghai University, Shanghai, China

**Keywords:** teacher-student relationship, coping style, sports anxiety, middle school students, physical education

## Abstract

**Objective:**

The present research aims to explore the relationship between coping style, teacher-student relationship, and sports anxiety among middle school students.

**Methods:**

A total of 289 Chinese middle school students from three schools in Shanghai participated in the survey.

**Results:**

(1) A negative teacher-student relationship significantly affects sports anxiety, and conversely, sports anxiety significantly impacts the negative teacher-student relationship, demonstrating a strong mutual effect; (2) Both active coping style and negative coping style significantly influence sports anxiety, while sports anxiety also significantly affects active coping style among middle school students. In the two-way model of teacher-student relationship (including positive and negative dynamics) and sports anxiety, the mediating effect of coping style is not significant.

**Conclusion and perspective:**

This study enhances our understanding of the roles of student-teacher relationship and sports anxiety, providing a concrete sample and example for educational authorities in developed countries to identify relevant issues in the field of physical education.

## 1 Introduction

Sports anxiety refers to the negative emotional reactions, including fear, tension, feelings of loss, and remorse, that individuals experience when faced with challenges in sports-related tasks, physical activities, or athletic competitions (Sangwan and Malik, 2024). In China, both schools and families tend to place excessive emphasis on intellectual and aesthetic education, which has led to a notable deficiency in sports values among teenagers. Furthermore, during the sensitive period for sports development, students often experience sports anxiety, characterized by significant psychological resistance to physical education classes and sports competitions (Bai, 2023). This phenomenon is driving adolescents away from sports participation. Furthermore, studies have indicated a rising prevalence of loneliness, depression, and suicidal behaviors among younger populations. This trend is closely associated with insufficient physical activity and limited participation in sports competitions during early adolescence (Wu et al., 2015; Tang et al., 2007; Liang et al., 2020; Huang et al., 2021). Importantly, sports anxiety is often a significant factor contributing to this phenomenon (Li et al., 2022).

In recent years, China’s Ministry of Education has implemented a series of policies regarding the high school entrance examination for physical education in Shanghai, with the objective of promoting sports participation among middle school students. Local governments are authorized to establish elevated standards that are tailored to their specific social and economic development needs. As one of China’s most economically advanced cities, Shanghai places a significant emphasis on sports development. Consequently, there is an urgent necessity to enact policies that enhance physical exercise among students by raising the physical education requirements for the high school entrance examination. Thus, the standards and expectations of the high school entrance examination for physical education in Shanghai are poised to serve as a crucial model for Chinese adolescents, encouraging improved physical training and active engagement in sports competitions. However, the heightened standards and rigorous requirements associated with the high school entrance examination for physical education in Shanghai may inadvertently intensify sports-related anxiety among junior high school students, potentially leading to increased feelings of frustration, avoidance, and emotional distress concerning their participation in physical activities and sports competitions.

Although China’s Ministry of Education has increased physical education classes in primary and secondary schools from two to three per week, this change has not significantly alleviated students’ sports anxiety. Concurrently, a lack of awareness regarding the value of sports among teenagers, coupled with the pressure of the high school entrance examination, has hindered the effectiveness of this increase in addressing students’ sports anxiety. Some scholars argue that, it is essential to explore solutions within the traditional teacher-student relationship to alleviate the sports anxiety experienced by junior high school students due to the emphasis on physical education in high school entrance examination (Experts Po, 2021). Respect for teachers and the valuation of education have long been foundational principles in Chinese educational culture. A positive teacher-student relationship, grounded in these values, is crucial for facilitating effective learning among adolescents (Ding et al., 2023). However, the prevailing perception of physical education as futile, coupled with the stigmatization of physical education teachers, often undermines the relationship between these educators and their students, contradicting traditional educational values. As a result, many physical education teachers struggle to maintain a positive outlook on their careers, which in turn exacerbates students’ sports anxiety.

Abroad study has highlighted the irreplaceable role of physical education teachers in managing students’ sports anxiety (Dolenc, 2015). A comprehensive analysis of the theories related to the influence of physical education teachers on students’ sports anxiety reveals that the attachment relationship between students and teachers often plays a pivotal role (Krstic, 2015). According to attachment theory, the strength of this relationship is closely linked to the development and manifestation of students’ anxiety levels (Wood, 2017). Teachers function as significant attachment persons for adolescents within the school environment and are frequently viewed as trusted mentors. As a result, adolescents’ attachment to their teachers can influence their anxiety levels (Dong et al., 2024), underscoring the importance of the teacher-student relationship as a factor affecting students’ sports anxiety. Furthermore, research suggests that the levels of sports anxiety experienced by students are intricately connected to their sports performance, which can, in turn, affect the teacher-student dynamic (Wood, 2017). This indicates that sports anxiety may negatively impact the interactions between teachers and students (Grisaffe et al., 2003). Additionally, the phenomenon of poor teacher-student relationships leading to the generalization of students’ fear suggests that anxiety originating from a single event may extend to multiple related situations, provoking further anxiety (Wang, 2015). Consequently, students experiencing sports anxiety may generalize these feelings, resulting in negative emotions toward their physical education teachers and ultimately impacting the overall teacher-student relationship. The aforementioned studies suggest a correlation and interaction between the teacher-student relationship and sports anxiety among middle school students. Accordingly, Hypothesis 1 (H1) posits that there exists a bidirectional predictive relationship between teacher-student relationship and sports anxiety experienced by middle school students.

The teacher-student relationship does not directly influence students’ sports anxiety; however, it can shape students’ psychology through various media, thereby affecting sports anxiety. The primary causes of sports anxiety include cognition, situational factors, and evaluation. In psychological terms, anxiety is categorized into two types: state anxiety and cognitive anxiety. The teacher-student relationship, as a cognitive factor contributing to the development of students’ state anxiety, is often a psychological stress reaction arising from the evaluation of the relational dynamics between teachers and students (Belcher et al., 2022). This psychological reaction is also referred to as coping style (Sabir, 2007). Coping is the cognitive and behavioral effort of individuals to consciously manage external or internal changes (Folkman, 2013), which can be divided into two types according to the ways of coping with problems: active coping and negative coping. Active coping refers to solving problems in a direct and rational way such as focusing on the positive and changing behaviors to solve problems and seeking social support (Ma et al., 2018), while negative coping refers to dealing with problems through avoidance, withdrawal and denial (Lin et al., 2020). According to Control Value Theory (Pekrun, 2006; Pekrun and Stephens, 2009), relational contexts can prompt the assessment of control and value, which is typically reflected in individuals’ subjective perceptions of control over their actions and their evaluations of their abilities. This, in turn, influences the accessibility of learning activities and processes through self-judgment (Pekrun, 2014). Coping style represents the psychological reaction and expression of this accessibility (Mcdonough and Ramirez, 2018). Research has indicated that the teacher-student relationship as perceived by students is significantly positively correlated with their sense of control and value (Zhang and Li, 2023). A heightened sense of individual control encourages individuals to adopt active coping styles, thereby reducing anxiety levels (Boals et al., 2011; Jian-Bin et al., 2016). Conversely, when students lack a sense of control in managing exercise-related situations, they are more likely to resort to negative coping styles, which can lead to increased sports anxiety (Basiaga-Pasternak, 2018). Therefore, Hypothesis 2 posits: coping styles may play mediation role in teacher-student relationships and sports anxiety among middle school students.

To summarize, this study aims to address two primary issues: (1) to investigate whether a correlation exists between the teacher-student relationship, a significant situational factor, and sports anxiety, as well as to determine if they can predict one another; and (2) to clarify the role of coping styles in the mediation of teacher-student relationships and sports anxiety. The findings of this research may contribute valuable principles for physical education in primary and secondary schools in China, thereby promoting the importance of effective teacher-student relationships in mitigating sports anxiety among junior high school students and encouraging the adoption of active coping strategies in response to sports-related anxiety.

## 2 Methods

### 2.1 Subjects and program

The additional physical education test for the high school entrance examination is designed to be applicable and equitable for all junior middle schools in Shanghai. This study, approved by the Ethics Committee of Shanghai University (approval number: ECSHU 2024–107), involved a survey conducted among third-grade students from three affiliated middle schools of Shanghai University. Informed consent was obtained from the students, their parents, and teachers, which included a detailed explanation of the study’s purpose, procedures, potential risks, and benefits. Questionnaires were distributed during regular class hours with the assistance of school teachers and volunteers who facilitated the session. All teachers and volunteers involved in the distribution received professional training that included detailed sessions on providing standardized instructions to participants, managing the distribution and collection of questionnaires, and monitoring the completion process to ensure consistency and reliability in test administration. A uniform instruction script was utilized across all sessions to minimize variation in participant guidance and ensure that all respondents received identical information regarding the completion of the questionnaire. To enhance student participation and encourage careful completion of the questionnaire, students who completed it meticulously were rewarded with athletic gifts as a token of gratitude for their participation in the survey. Additionally, all data collected were anonymized to maintain participant confidentiality, and any identifying information was securely stored and accessible only to the research team. A total of 289 students participated in the survey, resulting in the collection of 226 valid questionnaires. The respondents included 103 boys (45.6%) and 123 girls (54.4%), aged between 13 and 15 years.

### 2.2 Research tools

#### 2.2.1 Sports anxiety

This study integrated the SAS for 14-year-old Children (SAS2) developed by Smith et al. (2006) with the Adolescent Participation in Sports and Sports Anxiety Scale (PASAS) compiled by Norton et al. (2004) to assess the level of sports anxiety among middle school students. The scale encompasses four dimensions: emotional depression (e.g., “I do not want the ball to come to me when I play team sports”), attention disturbance (e.g., “I lose focus on the game”), physical anxiety (e.g., “My muscles feel tight because I am nervous”) and cognitive anxiety (e.g., “I feel that I will humiliate myself when I exercise/work out”). Responses were scored using 5-point Likert scale, where 1 indicates “completely inconsistent” and 5 indicates “very consistent.” A higher score reflects a greater degree of sports anxiety. The Cronbach’s *α* coefficient for sports anxiety scale among students in this study was 0.923.

#### 2.2.2 Teacher-student relationship

Based on the teacher-student relationship scale developed by Pianta (1994), this study utilized the Chinese version of the teacher-student relationship questionnaire adapted by Zhang (2003). The questionnaire comprised 22 items categorized into four dimensions: attachment (e.g., “I admire PE teachers very much”; “I always hope physical education teachers praise me in class”), intimacy (e.g., “I like physical education classes”; “physical education teachers work very hard to teach us sports movements”), conflict (e.g., “My opinions or suggestions are often ignored by physical education teachers”; “Many of my ideas are often misunderstood by physical education teachers”), and avoidance (e.g., “physical education teachers often make me feel nervous and uneasy”; “I do not want to take physical education classes”). The scale employed a 5-point Likert scoring system, where 1 indicated “strongly disagree” and 5 indicated “strongly agree.” In this study, attachment and intimacy were collectively referred to as the positive teacher-student relationship, while conflict and avoidance were collectively referred to as the negative teacher-student relationship. The Cronbach’s *α* coefficients for teacher-student relationship scale, the positive teacher-student relationship scale, and the negative teacher-student relationship scale in this study were 0.833, 0.856, and 0.946, respectively.

#### 2.2.3 Coping style

This study utilized the Simple Coping Style Questionnaire developed by Xie (1998) to assess coping styles according to the age characteristics and living habits of Chinese adolescents. The questionnaire contains a total of 20 items, which are scored using a 5-point Likert scale. 1 indicates “completely disagree” and 5 indicates “completely agree.” The instrument is divided into two dimensions: active coping, which includes 12 items (e.g., “I always stick to my positions and fight for what I want”), and negative coping, which encompasses 8 items (e.g., “I accept reality when I feel anxious in the face of difficulties, as there seems to be no alternative”). The Cronbach’s *α* coefficients for coping style scale, the active coping style scale, and the negative coping style scale in this study were 0.878, 0.885, and 0.714, respectively.

### 2.3 Data processing

Before conducting the statistical analysis, a common method deviation test was performed using SPSS 26.0. In this study, data were collected through paper-and-pencil questionnaires, utilizing several scales simultaneously; thus, differences among participants may exist. Therefore, common method deviation required attention. To control for potential common method deviation, the Harman single-factor analysis was conducted to test the possibility of systematic errors. The results showed that the variance explanation rate of the first factor without rotation was 15.6%, which is less than the 40% critical value, indicating that the common method deviation in this study was within acceptable limits. SPSS 26.0 was employed to conduct a reliability analysis of three scales, presenting Cronbach’s *α* for each scale, as well as descriptive statistics including mean scores and standard deviations (SD) for the three variables. Additionally, Pearson’s correlation analysis was performed. Amos 26.0 was utilized to carry out a confirmatory factor analysis (CFA) to assess the factorial validity of the three scales. Model fit was assessed using the *χ*^2^ test. A good model fit is indicated if *χ*^2^/*df* < 5, root mean squared error of approximation (RMSEA) ≤ 0.050, comparative fit index (CFI) ≥ 0.900, and Tucker–Lewis index (TLI) ≥ 0.900. The mediating effect serves to analyze the influence process and mechanisms through which independent variables affect dependent variables, establishing itself as an essential statistical method for examining relationships among multiple variables. In this study, the SPSS 26.0 PROCESS macro was applied to evaluate the significance of the mediating effect, while Bootstrap methods were employed to calculate mediation effect sizes. A total of 5,000 samples were generated, and a 95% confidence interval was calculated; the statistical results were deemed significant if the 95% confidence interval did not encompass zero.

## 3 Results

### 3.1 Descriptive statistics and correlation analysis

Pearson correlation analysis was employed to examine the relationships among teacher-student relationship, coping styles and sports anxiety in middle school students. The results are summarized in Table 1.

**Table 1:** Descriptive statistics and correlation analysis.^1^

	*M*	SD	1	2	3	4	5
1.SA	2.3921	0.68	1				
2.PTSR	3.9956	0.89	−0.129	1			
3.NTSR	1.4695	0.54	0.323**	−0.387**	1		
4.ACS	3.4274	0.69	−0.192**	0.162*	−0.144*	1	
5.NCS	3.0759	0.67	0.063	−0.024	−0.055	0.442**	1

^1^SA, sports anxiety; PTSR, positive teacher-student relationship; NTSR, negative teacher-student relationship; ACS, active coping style; NCS, negative coping style. **p < 0.01, *p < 0.05.

### 3.2 Difference analysis of demographic variables

Independent samples *t*-test was used to assess score differences between genders on each scale. The results showed no significant differences between boys’ and girls’ scores on all variables (Table 2).

**Table 2:** Gender differences in scores of each variable.

	Male (*n* = 103)		Female (*n* = 123)		t
	*M*	SD	*M*	SD	
1. SA	2.314	0.691	2.457	0.683	−1.563
2. PTSR	4.040	0.858	3.957	0.920	0.696
3. NTSR	1.446	0.547	1.488	0.552	−0.573
4. ACS	3.491	0.726	3.374	0.661	1.268
5. NCS	3.058	0.659	3.090	0.687	−0.359

### 3.3 Path analysis

Based on the research hypothesis, a structural equation model was established to analyze the influence of the teacher-student relationship on sports anxiety among middle school students. The CFI and TLI are both lower than 0.090, indicating that the model fit is suboptimal. By incorporating gender, sports participation and other relevant indicators as covariates for correlation analysis, the differences in the model did not change significantly. Consequently, the model was revised by evaluating the modification indices (M.I.) and changes in estimated parameters (Par Change). When covariant relationships between error pairs were identified, these errors were concatenated. By increasing the correlation path between the two residuals, the *χ*^2^ value of the model was reduced, and the modified model was re-estimated until it achieved an acceptable fit: CMIN/*df* = 1.045, *p* > 0.05, RMSEA = 0.014, CFI = 0.991, TLI = 0.989.

Through the model analysis of the two-way interactive relationship between teacher-student relationship and sports anxiety, it was found that the path results the impact of sports anxiety on teacher-student relationship did not meet the ideal fit standards, as both CFI and TLI were below 0.090. Therefore, the model was modified using a consistent approach, resulting in a model fit that met the required criteria: CMIN/*df* = 1.052, *p* > 0.05, RMSEA = 0.015, CFI = 0.989, and TLI = 0.987.

**Figure 1:**
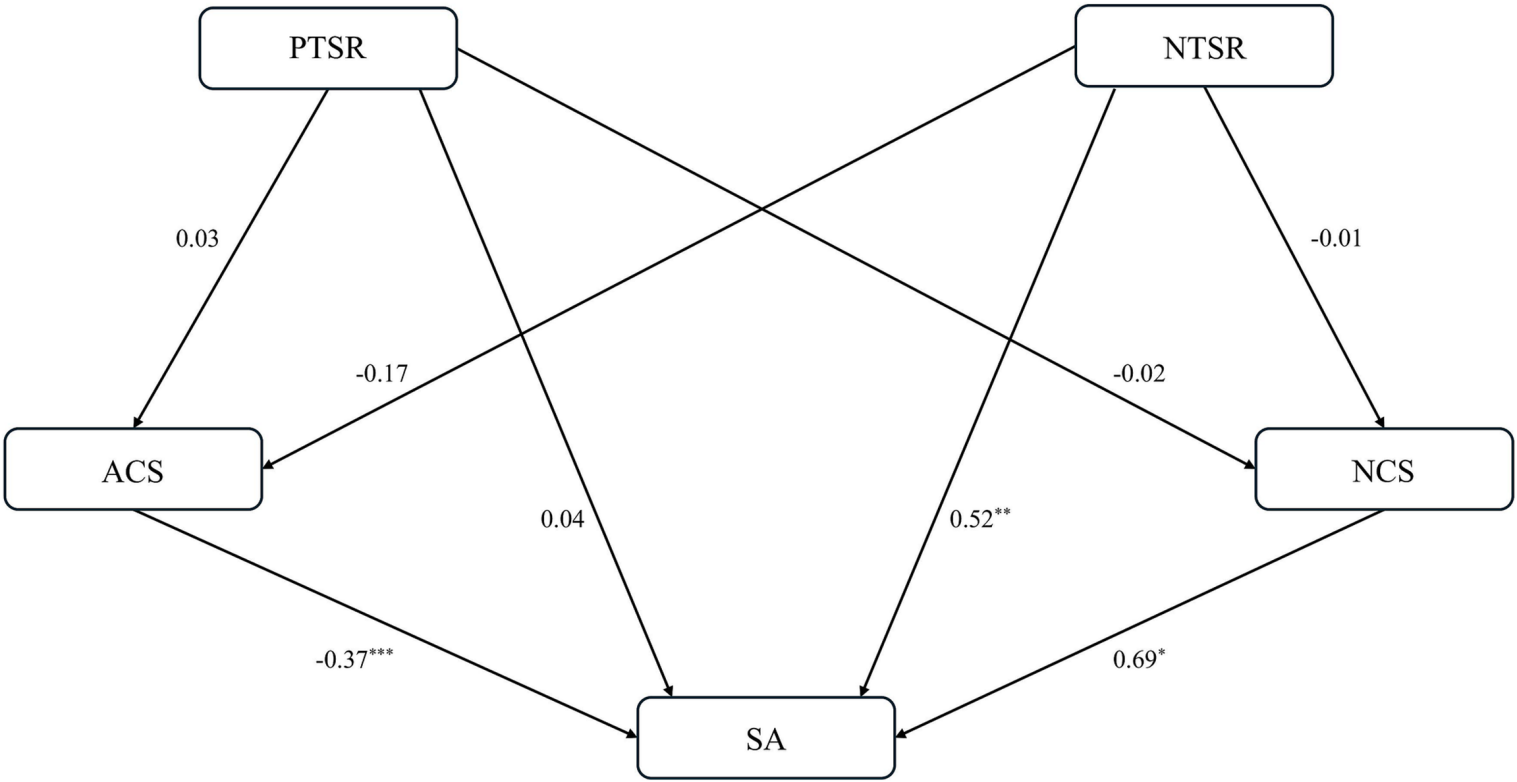
Path coefficients of PTSR/NTSR and ACS/NCS affecting SA. *p < 0.05, **p < 0.01, ***p < 0.001.

**Figure 2:**
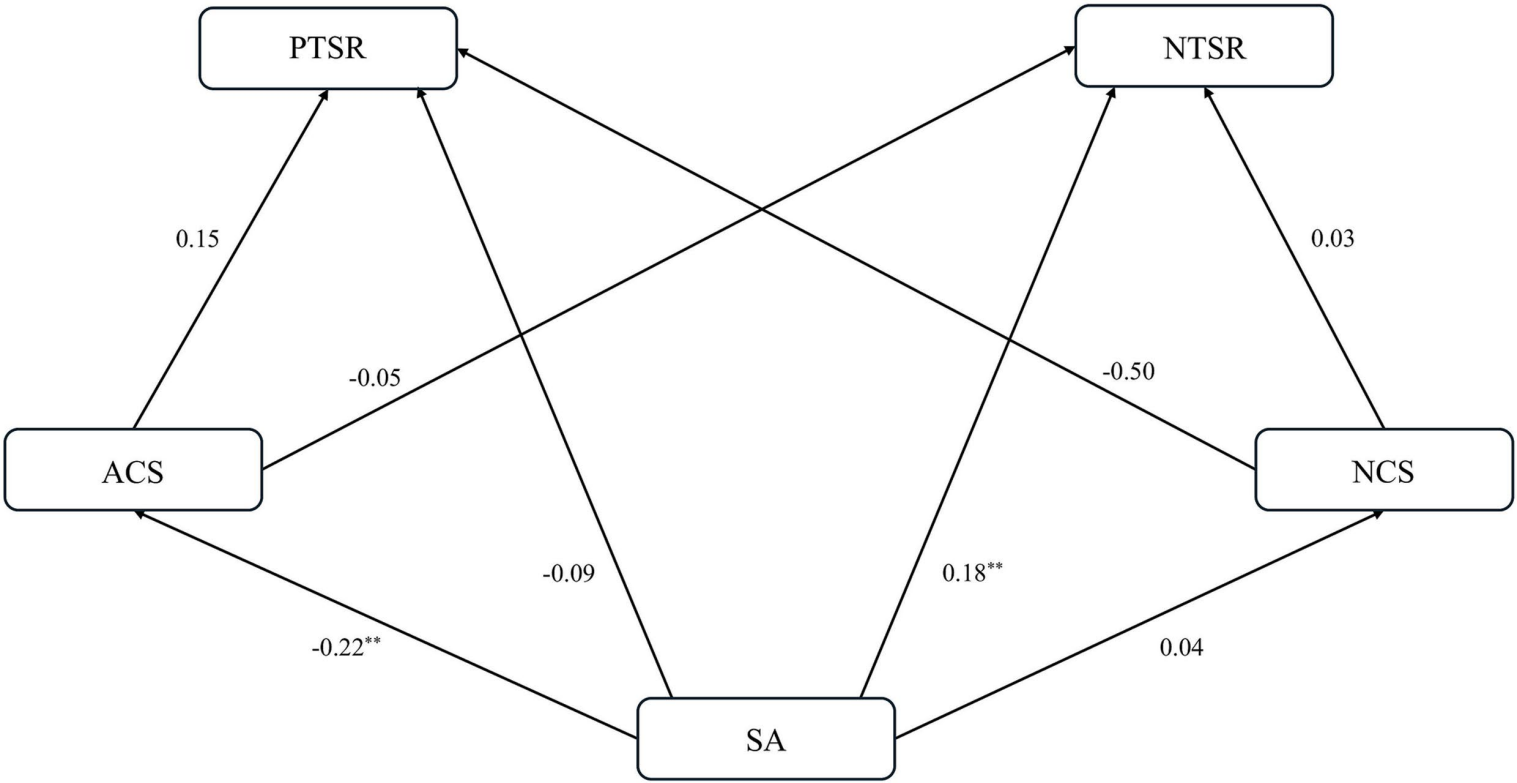
Path coefficients of SA affecting PTSR/NTSR and ACS/NCS. **p < 0.01.

The path results show that the interactive relationship between teacher-student relationship and sports anxiety is shown in Figures 1, 2. Specifically, the following relationships were identified: (1) NTSR → ACS → SA: (−0.17) × (−0.37) = 0.062, and (2) SA → ACS → NTSR: (−0.22) × (−0.05) = 0.011. Furthermore, path analysis of the model of how teacher-student relationship affects sports anxiety in the model showed that NTSR, ACS, and NCS had significant effects on SA [path coefficients were 0.52, (−0.37), and 0.69, respectively]. Conversely, in the model assessing how sports anxiety affects the teacher-student relationship, the path analysis demonstrated that SA significantly impacted ACS and NTSR, with path coefficients of (−0.22) and 0.18, respectively. In contrast, path analysis in a model examining how sports anxiety affects teacher-student relationship showed that SA significantly affected ACS and NTSR, with path coefficients of (−0.22) and 0.18, respectively.

Additionally, this study also tested the mediating effect of coping style, however, no mediating effect of coping style was observed (Table 3).

**Table 3:** Mediating effects of coping styles on teacher-student relationship and sports anxiety.

Path	Bootstrap (95%CI)	*p*	Indirect effect of standardization	Effect size
PTSR → ACS → SA	[−0.598, 0.001]	0.000	−0.011	−0.022
PTSR → NCS → SA	[−0.013, 0.016]	0.000	−0.013	−0.001
NTSR → ACS → SA	[−0.003, 0.035]	0.000	0.062	0.013
NTSR → NCS → SA	[−0.031, 0.009]	0.000	−0.006	−0.005
SA → ACS → PTSR	[−0.089, 0.005]	0.053	−0.033	−0.035
SA → NCS → PTSR	[−0.025, 0.024]	0.053	−0.020	−0.001
SA → ACS → NTSR	[−0.003, 0.035]	0.000	0.011	0.013
SA → NCS → NTSR	[−0.022, 0.005]	0.000	−0.001	−0.003

## 4 Discussion

Globally, the impact of early individual physical activity, games and competitions on the development of social cognition, awareness of rules, analytical and problem-solving skills, as well as the promotion of integrity and acceptance of future challenges, has garnered significant attention from scholars (Nopembri et al., 2019). However, due to the influence of cultural and educational factors, along with China’s large population, early youth development is often shaped by the educational perspective of “not losing at the starting line.” Consequently, the value of physical education has been overshadowed by academic competition from an early age, with participation in physical education and sports sometimes being labeled as “stigmatized.” Despite the government’s partial emphasis on the importance of physical education in the growth of young people within China’s early education system, physical education continues to face the educational dilemma of “marginalization” against the historical backdrop of the Chinese college entrance examination, where “10,000 people cross the one-tree bridge.” Furthermore, the low status of physical education teachers complicates the establishment of a positive cooperative relationship between teachers and students. A strong teacher-student relationship is crucial for alleviating students’ sports anxiety, and reducing this anxiety during competitions can enhance students’ sense of efficacy in engaging in sports, thereby fostering their interest in physical activities (Smith et al., 2007). With China’s rapid economic and social development, there is a pressing need to address the insufficient social attention given to youth physical education. To this end, physical education assessments must be integrated into examination results, thereby encouraging young people to recognize the importance of sports participation throughout their educational journey. However, the vital teacher-student interactions that should be fostered during the formative years of youth have been compromised, particularly in junior high school, where the focus on high school entrance examination has transformed these relationships into a challenging endeavor. Additionally, there is a notable lack of empirical research examining whether this resulting ambivalence correlates with sports anxiety among junior high school students. Should this association be substantiated, it would hold significant implications for dismantling social prejudices against physical education, advocating for the restoration of its value, and enhancing the physical well-being of millions of junior high school students. Furthermore, it could serve as a catalyst for reforming physical education in primary and secondary schools across developing countries.

Existing studies analyze the campus growth environment of adolescents from both objective and subjective perspectives. Objectively, supported by attachment theory, students exhibit a strong dependence on teachers throughout the educational process (Woolf, 2011). This dependence serves as a prerequisite for fostering interactive relationships, which, in turn, assist adolescents in identifying and resolving problems in a timely manner. This dependency becomes particularly pronounced when facing difficult and complex situations (Borup et al., 2013; Liu et al., 2006). Subjectively, emotional control theory suggests that adolescents’ ability to analyze and judge is increasing with the accumulation of knowledge during the educational process, and that could affect their choice of coping styles in response to situational changes (Jakobsen and Elklit, 2021). The present study investigated the mediating role of coping styles in the relationship between teacher-student relationship and sports anxiety among junior school students in a Chinese context. The findings indicate that negative teacher-student relationships significantly impact students’ sports anxiety. Furthermore, sports anxiety also significantly influences the quality of teacher-student relationship, highlighting a strong mutual effect. These results are supported by the findings of the current study (Salter et al., 2024); at the same time, both active and negative coping styles significantly influence middle school students’ sports anxiety. Moreover, sports anxiety significantly affects active coping styles, the finding that is corroborated by the existing literature (Anshel and Sutarso, 2007). In the bidirectional model where teacher-student relationships (both positive and negative) impact sports anxiety and vice versa, the mediating effect of coping styles was not found to be significant. This lack of a significant mediating effect contrasts with the findings of the current study (Spilt and Koomen, 2022), highlighting the unique characteristics of junior school students’ coping styles in relation to teacher-student relationship and sports anxiety within the Chinese context.

According to attachment theory, a positive teacher-student relationship serves as the foundation for effective relation between teachers and students (Ye and Pang, 2001) and mitigates students’ academic anxiety (Kurdi and Archambault, 2018). However, the findings of this study indicate that positive teacher-student relationships do not significantly alleviate students’ sports anxiety, which contradicts existing research. In contrast, negative teacher-student relationships appear to exacerbate the development of students’ sports anxiety. This indicates that the impact of teacher-student relationships on sports anxiety may be context-specific, particularly within the Chinese educational framework. The observed contradiction may stem from the social ‘stigmatization’ of physical education teachers and the ‘marginalization’ of middle school students’ values related to sports. This further reinforces the idea that the establishment of positive teacher-student relationships in physical education is not universally applicable at this stage of social development (Bai, 2023). Therefore, it is crucial for China’s education authorities to thoroughly consider the potential implications of early physical education within the national education system. This perspective also implies that sports anxiety should not be regarded as a counteractive mechanism against the benefits of positive teacher-student relationships. Conversely, the discordant relationships between physical education teachers and students contribute to heightened levels of sports anxiety among students, a phenomenon that aligns with the principles of attachment theory. Research suggests that when physical education teachers impose specific requirements on students regarding exercise time or intensity, students may perceive these demands as burdens, which can strain the teacher-student relationship and intensify sports anxiety (Zu et al., 2009). Although existing literature generally supports a positive correlation between a negative teacher-student relationship and increased student anxiety (Baker et al., 2000), it is important to acknowledge that, due to the delayed development of physical education in China, the causes and mechanisms underlying this relationship may differ from those observed in other educational settings. Therefore, it is crucial to fundamentally reshape our understanding of physical education in early childhood. Initiatives aimed at fostering a social atmosphere that values physical education, allocating more time for youth physical activity, and increasing financial investment in facilities are crucial measures. These efforts will enhance the teacher-student relationship in physical education, promote the development of physical activity habits among youth, and strengthen their commitment to engaging in physical activity (Chai and Wu, 2023; Guo et al., 2023; Sun, 2022).

In this study, coping styles did not mediate the relationship between teacher-student interactions and sports anxiety. Rather, the development of sports anxiety is a chain reaction of psychological mechanisms that includes both trait anxiety and state anxiety, particularly in the context of sports and competitions. Existing literature explains sports anxiety through emotion control theory, suggesting that Chinese adolescents often lack opportunities for physical activity during the initial phases of physical education (Wu et al., 2023). Furthermore, they have not been exposed to quality physical education throughout their developmental years. Additionally, inadequate community sports facilities and insufficient family education regarding physical education and health promotion contribute to a general inability to understand the functions and values of sports through active participation, thereby exacerbating the formation of sports anxiety. As a result, students are prone to sports anxiety when faced with the compulsory demands of sports or competitions in junior school, yet they often lack appropriate coping strategies. Sports anxiety, as an emotional manifestation, is closely linked to adolescents’ self-regulatory behaviors, and active coping styles are cultivated through the beneficial experiences gained during prolonged sports participation. The emergence of negative coping styles among contemporary Chinese adolescents is not solely a product of their subjective experiences; rather, it is also influenced by the absence of a well-defined network of sports values within Chinese society, which significantly impacts adolescents’ negative coping mechanisms (Su et al., 2019). Studies have shown that active coping styles help prevent anxiety, while negative coping styles may exacerbate anxiety (Baker et al., 2000; Cardozo et al., 2013; Li, 2022; Yan et al., 2021). This is consistent with the results of this study that active coping style reduces sports anxiety while negative coping style increases sports anxiety. At the same time, previous research has indicated that adolescents who engage in early and sustained sports and competition tend to develop a heightened perception of emotional control and exhibit more active coping styles. Conversely, a lack of involvement in sports and competitions is associated with negative coping styles. This absence of sports participation contributes to the prevalence of sports anxiety among Chinese adolescents, where both trait anxiety and state anxiety emerge as a result of limited engagement in sports and competitions, often manifesting as intentional avoidance of these activities (Bu et al., 2017), and active distancing from physical education and extracurricular sports activities competitions (Zhong and Liu, 2024). At a deeper level, this lack of engagement affects the establishment of positive teacher-student relationships. Previous studies have shown that the development of adolescents’ coping mechanisms for sports anxiety is significantly influenced by the quality of teacher-student relationships (Cho, 2017). In traditional Chinese educational values, respect for teachers forms the relational bond that fosters students’ attachment to their educators (Yang, 2024). In this context, physical education teachers are more likely to convey educational values in a commanding and accepting manner, and are less inclined to encourage students to identify and solve problems independently (Zhang and Chen, 2007). Consequently, the formation of active coping styles becomes even more challenging, particularly given that the values of physical education have yet to be fully integrated into the national education system. With the ongoing reform and development of education in China, the explicit value of physical education is gradually being recognized. The introduction of additional examinations for physical education in high school entrance examination serves as a mechanism to challenge the entrenched perceptions of this discipline. The concept of a physically educated nation is steadily taking root in the public consciousness, and national fitness has been elevated to a national strategy. Collectively, these policies and initiatives are fostering significant reforms in physical education within Chinese schools. Notably, the product of coefficients approach of path analysis is commonly used to assess mediation effects in this study, the presence of non-significant mediation roles should be interpreted cautiously and may do not provide definitive evidence of mediation.

Of course, the limitations of this study are evident. The adolescents’ understanding of sports varies significantly, which presents factual challenges for the survey. Additionally, to facilitate the research, the questionnaire was administered to students from three middle schools in Shanghai, resulting in a limited sample size that may affect the scientific rigor of the findings. Furthermore, the survey primarily targeted students preparing for the physical education extra examination associated with high school entrance examination. This focus may hinder the students’ ability to fully comprehend their past experiences in physical education, particularly regarding the influence of teacher-student relationships on sports anxiety. Often, their responses reflect only the pressures associated with the physical education extra examination. As a specific form of sports competition, the extra exam elicits a short-term emotional response to students’ sports anxiety, making it challenging for the establishment of a supportive teacher-student relationship to provide immediate coping strategies. Consequently, fostering a positive teacher-student relationship in early physical education is essential for alleviating adolescent sports anxiety and encouraging constructive coping mechanisms, which is the primary objective of this study. Additionally, long-term effects of different coping strategies or their applicability across diverse populations can be incorporated into the future study.

## 5 Conclusion

This study demonstrates the relationship between teacher-student relationships and sports anxiety and the role of coping styles in this process. Participation in sports activities and competitions is an essential component of individual socialization and emotional education worldwide (Sinha, 2024). Countries often emphasize the importance of students’ integration into society through sports participation in basic education (Seippel, 2018). Unified values have been established at all levels—nation, community, school, and family—and are implemented through specific actions (Van Boekel et al., 2016). Although China’s rapid economic development has greatly increased people’s awareness of the value and role of physical education in the national education system, establishing an effective and operational pathway for physical education from planning to implementation remains a challenging task (Li, 2016).

The relevant conclusions of this study underscore the importance of recognizing the value of physical education in shaping the future of high-quality basic education (Liu et al., 2014). It is crucial to enhance the status and sense of belonging of physical education teachers within the school settings (Allen et al., 2021) and to effectively encourage students to actively engage in sports activities (Liu and Ding, 2011). Additionally, it is important to explore solutions to sports-related anxiety by emphasizing the value of participation in sports. This approach can improve students’ coping strategies during sports activities and promote the overall development of physical education.

## Data Availability

The raw data supporting the conclusions of this article will be made available by the authors, without undue reservation.
